# Classification of Public Health Centres in Accra through a Web-Based Portal Integrated with Geographical Information System (GIS)

**DOI:** 10.1155/2021/4178161

**Published:** 2021-12-03

**Authors:** Nana Yaw Asabere, Gare Lawson, Godwin Badu-Marfo, Lydia Kwofie, Daniel Opoku Mensah, Reginald Lartey

**Affiliations:** ^1^Department of Computer Science, Faculty of Applied Sciences, Accra Technical University (ATU), Accra, Ghana; ^2^DexAfrica Limited, Accra, Ghana; ^3^Department of Geography Urban Planning and Environmental Studies, Concordia University, Montreal, Canada

## Abstract

A health system is described as a logically organized collection of resources, agents, and institutions that offer healthcare to a specific population based on the finance, regulation, and delivery of health services. Many health centres have been established in Accra, the capital city of Ghana, due to the importance of good health. People in other developed nations can seek adequate healthcare, since information about relevant health centres is readily available. However, there is a paucity of information about the services provided by existing health institutions in Ghana, particularly in Accra. The majority of patients commute to either Korle-Bu Teaching Hospital or Greater Accra Regional Hospital, putting a considerable medical strain on these facilities. In this study, we use a Geographic Information System (GIS) to establish a database for all of Accra's health centres and categorize them according to the services they provide. This research tackled the previously mentioned problem by proposing and developing a web-based map called *Geohealth* for the classification of public health centres in Accra using GIS to assist users in accessing information and locating health centres. We utilized a mixed-method approach consisting of quantitative as well as Build Computer Science Research Methods. Results of our study show that the majority of the participants and stakeholders in our research are eager to embrace *Geohealth*. Furthermore, in comparison with existing techniques such as Google Maps, our proposed approach, *Geohealth*, takes less time to obtain information and locate public health centres in Accra, Ghana.

## 1. Introduction

Health is defined by the World Health Organization (WHO) as “a condition of complete physical, mental, and social quality of life, not only the absence of sickness or disability” [1]. According to Ramzi and El-Bedawi [2], effective delivery of healthcare critically requires allocating resources, economic growth, and the geographic distribution of inhabitants and communities across a country, as well as interconnectedness and accessibility from urban regions. However, in Africa, existing spatially explicit population data, on the other hand, are mostly based on obsolete and low-resolution input demographic data. Consequently, the required details to quantify rural settlement patterns are insufficient [3].

In developing nations, systematic healthcare facilities and accessible systems are largely centered in metropolitan regions rather than in rural areas, causing inconvenient access to healthcare centres for low- and middle-income citizens [4]. Generally, driving and walking (especially in rural areas) are the two main commutation procedures used to arrive at a health centre.

Mansour [5] highlighted that the basic concept of health utility delivery inside larger cities is the dispersal of health facilities in metropolitan regions. Exploring and assessing the geographical link between health centre sites and geographic accessibility to such centres has been an essential element for decision-makers, planners, and healthcare systems for a long time [6].

Quite a number of studies on Geographical Information Systems (GIS), healthcare, and health centres have been conducted. For example, dos Anjos and Cabral [7] utilized health facilities' coordinates together with demographic, elevation, and auxiliary data to simulate accessibility to Health Centres (HC) using GIS, according to their research on geographical accessibility to primary healthcare centres in Mozambique. Similarly, Edward and Biddle [8], Garcia et al. [9], and Ravitch et al. [10] also used GIS to solve health-related issues such as geospatial analysis and geographic accessibility of healthcare. However, the aspect of categorizing services offered by specific health centres is not available in any of these studies. The services provided by a health facility are very important to avoid a situation of a patient arriving at a health centre which cannot provide emergency and immediate services required.

As a result of the above, this paper seeks to utilize GIS to develop a web map of all public health centres in Accra and categorize them according to the services they provide. Our proposed computerized web-based map extracts, transforms, and loads data or information onto a platform using GIS capabilities. Based on the data collected throughout the study and subsequent integration of the web-based map, this technology can be used to classify public health facilities in Accra.

This paper aims to provide the end-user (who at this stage may be a patient) with accurate and timely information on the healthcare services provided by Accra's public health institutions. Furthermore, the paper aims to address the problem of not having enough information about a health centre's services as well as the location of the health centre to be visited. The paper is chronologically structured as follows: In Section 2, we review the main concept of GIS, as well as some related works and the theoretical framework that we employed in our research. We further evaluate our data using descriptive inferential statistics after analyzing it with SPSS in Section 3, which is devoted to the study methodology, data collection, and analysis. In Section 4, we present our proposed *Geohealth* solution. In Section 5, we conclude the paper with some details of future work.

## 2. Review of Literature

This section includes an overview of the literature and its components. The literature evaluation of a web-based map for classification of public health institutions in Accra using GIS is focused on essential sections such as (i) GIS definition and concept, (ii) GIS in healthcare, with a focus on public health centres, (iii) GIS in healthcare in Africa and Ghana, (vi) Related work and perspectives of GIS researchers, and our adopted Theoretical Framework. Our literature review, therefore, focused on research publications linked to GIS concerning public healthcare and health centres.

### 2.1. Definition of GIS

We employ a wide definition based on an information system that manages geographic, spatial, or geospatial data for spatiotemporal usage and geographic research. GIS is a conceptual framework for capturing and analyzing spatial and geographic data. [11, 12]. GIS allows a user to compare and contrast a variety of various sorts of data. Data regarding people, such as population, income, and education level, as well as the geographic position of physical structures, may all be included in the system. It can include information on a terrain, such as stream locations, building locations, plant types, and soil types [11, 12].

### 2.2. GIS in Healthcare

GIS and related spatial analytic approaches provide a set of tools for documenting and analyzing the evolving geographical structure of healthcare, investigating its relationship to health access, and evaluating how healthcare might be delivered more effectively [13]. How GIS may be used to analyze healthcare needs, evaluate, plan, and access healthcare service locations, and enhance spatial decision-making in healthcare has been explored. Access to integrated geographical data on health services use and outcomes associated with human service systems will be required for healthcare researchers and policymakers to utilize GIS [14]. Healthcare encompasses a wide range of services, from personal health services to health education and information for illness prevention, early detection, treatment, and rehabilitation [15]. The importance of GIS is appropriate for the objective of modern public health, which is defined by the WHO as “the attainment of the best attainable level of health by all people” [16].

### 2.3. GIS in Healthcare in Africa and Ghana

A health system's proximity to a health facility is a significant factor. It has an impact on the illness load, which mostly affects poorer nations, particularly Africa. As a result, measuring proximity has an impact on the health system's performance and contributes to policy reform. Tanou and Kamiya [17] emphasized that despite the formal establishment of the universal National Health Insurance Program (NHIS) and Community-Based Health Planning and Services (CHPS) in Ghana since 2003, the majority of Ghanaians do not appear to have geographic access to healthcare. In the case of many developing nations, improving mother and child health (MCH) is still a major problem. The distance between a person's home and the nearest health facility is seen to be a major impediment to the utilization of adequate Maternal and Child Health (MCH) services, particularly in Sub-Saharan African nations. Women's utilization of adequate healthcare services during pregnancy and delivery is still low in Burkina Faso, a landlocked country in West Africa's Sahel area. As a result, the influence of geographic proximity to health facilities on maternal healthcare usage in Burkina Faso was investigated in [18].

Ghana's government has implemented the Community-Based Health Planning and Service (CBHPS) initiative to alleviate regional service disparities within the country. This is designed to improve access to services in rural and underserved regions by refocusing the community's attention on primary health care (PHC). CBHPS zones were established with some having a physical structure (CHPS compounds) for service provision and staffed by paid community health nurses, while others had none [19].

Efforts to enhance the operation of health systems need spatial data that visualizes the true distribution of illness burden. Geographic mapping enhances resource deployment prioritization by identifying areas where certain issues are concentrated. In recent decades, there have been widespread agreements that GIS technology is the most effective instrument for gathering, storing, and displaying retrievable data. This enables managers to monitor, evaluate, and target regions where resources are best allocated [20].

### 2.4. Related Work in GIS for Healthcare

In this section, we review existing GIS research in healthcare in connection to health centre classification in the literature to support the idea of a computerized web-based map for the categorization of public health facilities in Accra.

GIS mapping, for example, was utilized by Edward and Biddle [8] to identify high-need regions for access to primary healthcare. The location of providers and their distance from patients are regarded as the major obstacles to treatment when designing interventions to enhance access to primary healthcare. Consequently, they critically incorporated spatial aspects in their work and employed tools to assess the geographical border that has been defined.

Garcia et al. [9] utilized geospatial analysis to evaluate the obstacles to healthcare access among a specific population of immigrants. They concluded that spatial variables such as the location of healthcare institutions and transportation difficulties are creating barriers to healthcare access.

Ravitch et al. [10] examined the geographic accessibility of pediatric asthma physicians. They discovered that the health outcomes of these patients differed by location and that this was linked to the amount of access to treatment as well as other demographic factors such as the patients' education and income level. Consequently, they posited that locations with a shortage of physicians and low-income families should be targeted to enhance the health outcomes of pediatric asthma patients.

In a related study, dos Anjos and Cabral [7] showed that the use of GIS in public health has grown dramatically as a result of the availability of various information technology services and software and that it is now considered useful in the understanding and treatment of health problems in various geographic areas.

The related studies above illustrate that GIS has been utilized in the area of healthcare in terms of spatial processes, geospatial analysis, geographic accessibility, and assessing geographic borders. However, there is currently a lack of location-based GIS combined with specific services offered by health centres which are extremely important for healthcare delivery. We, therefore, seek to fill this gap using the public health centres in Accra as our target data.

### 2.5. Theoretical Framework

A theoretical framework is a key component of any research project that attempts to improve knowledge of observable events or offer a lens through which they may be evaluated. The theoretical framework, according to Venkatesh et al. [21], aids researchers in defining the goal of a study and its contribution to the body of knowledge. It is critical to use the proper model or theoretical framework to explain numerous processes and procedures related to technological acceptance. Depending on the scientific topic of research, accessibility can be characterized in a variety of ways [21].

Venkatesh et al. [21] developed the Unified Theory of Acceptance and Use of Technology (UTAUT) model. The model was created by examining and validating eight existing hypotheses that can predict behavioral intentions to utilize information technology. Theory of Reasoned Action (TRA), Technology Acceptance Model (TAM), Motivational Model (MM), Theory of Planned Behavior (TPB), a combined Theory of Planned Behavior and Technology Acceptance Model (TPB-TAM), Model of Personal Computer Use, a combined Theory of Diffusion of Innovations Theory (MPC-TDIT), and Sociocognitive Theory (SCT) are the eight models currently available [21].

**Figure 1:**
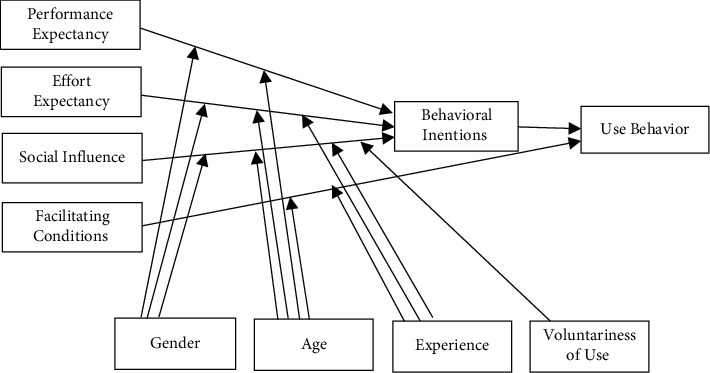
UTAUT model.

Figure 1 depicts the UTAUT model, which includes the components and their relationships. Our adoption of the UTAUT is substantiated by the fact that it is believed to be a robust contemporary predictive theory for behavioral intention to accept and use information technology [21].

Venkatesh et al. [21] compared the UTAUT model to the eight existing models and found that the UTAUT surpassed them all, accounting for 70% of the variance in behavioral intention (BI) and roughly 50% of the variance in actual usage. The UTAUT components, that is, Performance Expectancy (PE), Effort Expectancy (EE), Social Influence (SE), and Facilitation Conditions, were created as a result of the unification of the eight models.

## 3. Materials and Methods

This section describes the techniques used in the research study to collect and obtain appropriate data for successful analysis. This section contains information about the study's population and sample, as well as the data collection instrument and findings of the study after data analysis.

### 3.1. Research Methodology and Data Collection Instrument

The research technique used in this study was a hybrid (mixed-method) strategy that included Quantitative and Build Computer Science methodologies. To validate the proposed system, the Build Computer Science (software development) technique was used. In addition, the quantitative method was used to confirm the importance of technological adoption in constructing our proposed web-based map for the classification of Accra's public health institutions. The quantitative method which involved the administration of questionnaires through an online Google Form was selected due to the benefit of reaching out and obtaining more information from a large number of people who are not situated in one place.

**Figure 2:**
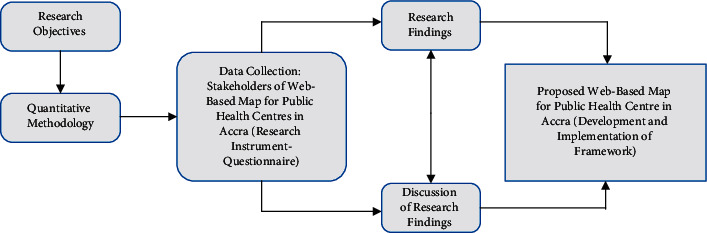
Research process.

Figure 2 depicts our research process. The questionnaire was sent out to residents of the Greater Accra Region to determine the feasibility of using technology to create a web-based map for Accra's public health centres. The questionnaires were sent out to about 500 research participants using online Google Forms. Each respondent received a questionnaire with thirty main questions and seven subdivisions, including Demography (three questions), *Geohealth* Acceptance as a tool for accessing health care (ten questions), Technology Performance Expectancy (three questions), Technology Effort Expectancy (four questions), Social Influence on Technology (two questions), Behavioural Intention (four questions), and Facilitating Condition (four questions).

### 3.2. Population and Sample of Study

Residents of the Korle-Klottey area were the participants involved in our study. Before becoming a district, the Korle-Klottey area was a submetro of the Accra Metropolitan Assembly. Korle Gonno, Korle-Bu, Chorkor, Mamprobi, New Mamprobi, and James town are all part of the Korle-Klottey area. The Korle-Klottey district is divided into several villages. Because participants were chosen based on their availability, location, and desire to participate, we opted to utilize the purposively sampling approach. The settlements of Osu, Korle-Bu, and Chorkor were chosen as the sample population because they are located near several of Accra's public health centres, which serve the vast majority of the population.

The Korle-Klottey district was chosen at random for respondents. As a result, a total target population of 650 individuals (*N* = 650) was established. A total of 280 online surveys were sent out to participants. We obtained 260 responses from the participants, which equates to a response rate of 92.9 percent. This response rate indicates that we received a sufficient number of surveys to conduct data analysis.

We used a common mathematical equation from Kothari [22] to assure the reliability and validity of our sample size of the respondents, as indicated in the following equation:

(1) $$\begin{array}{c} n=\frac{Z^{2}\times p\times q\times N}{e^{2}\times \left(N-1\right)+Z^{2}\times p\times q}, \end{array}$$

where *n* is the sample size, *Z* is the confidence level, *p* is the probability of success, *q* = 1 − *p*, *N* is the population, and *e* is the precision level. As a result, we used (1) to achieve our anticipated sample size (*n*). In order to calculate *n*, we used the following values in (1): *Z* = 1.96, *p* = 5% (0.05), *q* = 1–0.05 = 0.95, *N* = 650, and *e* = 0.02. Equation (2) illustrates this calculation:

(2) $$\begin{array}{c} n=\frac{1.96^{2}\times 0.05\times 0.95\times 650}{0.02^{2}\times \left(650-1\right)+1.96^{2}\times 0.05\times 0.95}=\frac{118.61}{0.442}=268.35. \end{array}$$

As shown in (2), our computed sample size was 268.35 (*n* = 268.35). For the analysis to be effective, we had to receive responses close to 268.35 or more. Regarding the sample size, we received 260 responses and therefore used these responses for effective data analysis as shown in the tabulated results below. The quantitative data was analyzed using the Statistical Package for Social Sciences (SPSS). Results of our data analysis using SPSS are presented below in descriptive statistics and percentages.

## 4. Result of the Study

The sections below present the results of the study using descriptive inferences and statistics.

### 4.1. Demography and Profile of the Respondents

Table 1 illustrates the demographic profile of respondents. Our study involved a total of 260 participants: 160 (61.5%) males and 100 (38.5%) females. The majority of the participants (113) in this study are in the age group of 25–34 years (43.5%), followed by 64 participants in the age group of 45–54 years (24.6%), 44 participants in the age group of 15–24 years (16.9%), 38 participants in the age group of 35–44 years (14.6%), and one participant in the age group of 55 and above (0.4%).

Further information on respondents' Technology Acceptance, Technology Performance Expectancy (TPE), Technology Effort Expectancy (TEE), Social Influence (SI), Facilitating Condition (FC), and Behavioral Intention (BI) is available in the sections below. TN = Total Number, *M* = Mean, SE = Standard Error, SD = Standard Deviation, and *V* = Variance are the statistical representations shown in the tabulated results below.

A high *M* value in our data analysis reflects the most popular category among the participants. Furthermore, a low SE equates to a high *M*. As a result, if the SE for a given category is large, *M* for that category is invalid. Furthermore, SD denotes the dispersion of the obtained data, whereas *V* denotes the variations in the mean of the Likert scale for a certain category.

### 4.2. Existing Situation and Technology Acceptance of Respondents

In relation to technology acceptance, the greatest *M* value (*M* = 1.77, SE = 0.03, SD = 0.42, *V* = 0.18) relates to participants confirming that they did not find it easy to identify the health centre they wanted to attend, according to the data in Table 2. Participants then stated that the health clinics they visited did not provide the services they requested (*M* = 1.73, SE = 0.03, SD = 0.44, *V* = 0.20).

Furthermore, data from Table 2show that individuals were admitted to a health centre for treatment at the next level of a high*M* (*M* = 1.10, SE = 0.02, SD = 0.31, *V* = 0.09). Additionally, the participants confirmed that they did not receive a bed assignment soon after admission (*M* = 1.57, SE = 0.03, SD = 0.50, *V* = 0.25) and that the health centres had adequate capabilities to handle their condition (*M* = 1.39, SE = 0.03, SD = 0.49, *V* = 0.24).

Furthermore, the results in Table 2 show that the majority of participants have been turned away by a health centre they visited because it does not provide the services they require (*M* = 1.27, SE = 0.03, SD = 0.45, *V* = 0.20), followed by participants indicating that they would accept *Geohealth* as a system for accessing information and location about health centres (*M* = 1.03, SE = 0.01, SD = 0.16, *V* = 0.03), as well as the availability of smartphone to aid their access or usage of *Geohealth* by participants (*M* = 1.00, SE = 0.00, SD = 0.06, *V* = 0.00).

In summary, the respondents' technological views on access to health centres and accepting *Geohealth*, as shown in Table 2, validate that if a system like *Geohealth* is implemented, they would appreciate the use of technology to facilitate their access to information and the location of public health centres.

### 4.3. TPE of Respondents

In terms of TPE, the greatest *M* value (*M* = 1.10, SE = 0.02, SD = 0.31, *V* = 0.09) relates to participants confirming that they will find *Geohealth* beneficial and efficient in visiting health services (see Table 3). Participants then stated that utilizing *Geohealth* would allow them to get healthcare services more quickly and easily (*M* = 1.10, SE = 0.02, SD = 0.30, *V* = 0.09). According to participant responses, one of the most important aspects of utilizing *Geohealth* would be efficiency and time management, as it will allow people to check health facilities closest to them and their services before visiting (*M* = 1.09, SE = 0.02, SD = 0.29, *V* = 0.08).

### 4.4. TEE of Respondents

As shown in Table 4, the greatest *M* value in terms of effort expectation (*M* = 1.11, SE = 0.02, SD = 0.31, *V* = 0.10) substantiates that, in relation to participant answers, *Geohealth* will not be as difficult to use (see Table 4). Participants agree that learning to use *Geohealth* will be simpler for them (*M* = 1.10, SE = 0.02, SD = 0.31, *V* = 0.09).

In addition, the results in Table 4 demonstrate that participants' interactions with *Geohealth* are clear and understandable in terms of their wellbeing (*M* = 1.10, SE = 0.02, SD = 0.31, *V* = 0.09) and that learning to use *Geohealth* is simple (*M* = 1.10, SE = 0.02, SD = 0.30, *V* = 0.09).

### 4.5. SI of Respondents

In relation to the responses from participants in Table 5, the greatest *M* value (*M* = 1.14, SE = 0.02, SD = 0.35, *V* = 0.12) substantiates that a person who influences participants' behavior would recommend that they utilize *Geohealth*, as shown in Table 5. Finally, individuals they care about believe they should use *Geohealth* because it is beneficial to them (*M* = 1.12, SE = 0.02, SD = 0.32, *V* = 0.10).

### 4.6. FC of Respondents

As shown in Table 6, the facilitating condition with the greatest *M* value denotes that*Geohealth* is not accessible on the browser they use (*M* = 1.66, SE = 0.03, SD = 0.48, *V* = 0.23), followed by the fact that assistance is available if they have any problems using *Geohealth* (*M* = 1.16, SE = 0.02, SD = 0.37, *V* = 0.13).

Furthermore, participants said that they have the resources required to utilize *Geohealth* (*M* = 1.15, SE = 0.02, SD = 0.35, *V* = 0.13) and that they have the knowledge required to use *Geohealth* (*M* = 1.14, SE = 0.02, SD = 0.35, *V* = 0.12).

### 4.7. BI of Respondents

In relation to the responses from participants in Table 7, the greatest *M* value (*M* = 1.10, SE = 0.02, SD = 0.30, *V* = 0.09) indicates that participants expect to utilize *Geohealth* in the future in terms of behavior. Participants predicted they would use *Geohealth* in the future (*M* = 1.10, SE = 0.02, SD = 0.31, V = 0.09), which led to this study.

Additionally, participants also want to suggest *Geohealth* to relatives and friends (*M* = 1.10, SE = 0.02, SD = 0.31, *V* = 0.09) and plan to use *Geohealth* in the future (*M* = 1.09, SE = 0.02, SD = 0.29, *V* = 0.08) at the next level. The results shown in Table 7 show that the majority of responders via BI are prepared to adopt technology, paving the path for *Geohealth*.

The respondents are prepared to adapt and accept our proposed web-based map for obtaining information and the location of public health centres in Accra, based on the tabulated analysis and results illustrated above. Consequently, we elaborate on our proposed method in the next section.

## 5. Proposed Solution: *Geohealth* for Public Health Centres

**Figure 3:**
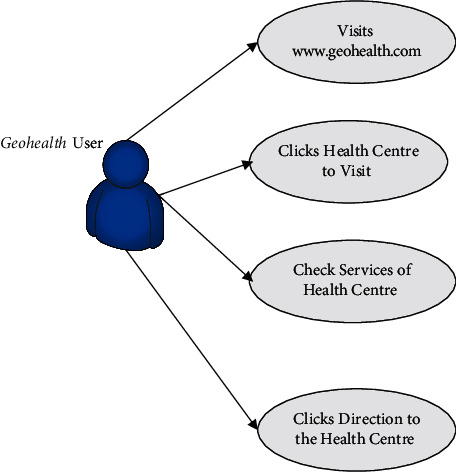
Use case diagram of Geohealth.

In this section, we describe our proposed *Geohealth* system. In our proposed method, GIS and public health data in Accra, Ghana, are integrated so that *Geohealth* as a web-based map can be used by public health facilities.  Figure 3 illustrates the use case diagram for *Geohealth*. *Geohealth* is an innovative approach to acquiring information on some of Accra's health centres that are not available through regular means. Web-based mobile and desktop systems will be used to deliver *Geohealth* services.

### 5.1. Functional Requirements

The functional requirements identified are the following:

- The system will help the user acquire accurate information on the intended health centre to visit.
- The system will help users obtain updated information about the services rendered by the various health centres.
- The system would be able to find information about the number of users who have accessed the system and have efficiently utilized the tools.

### 5.2. Nonfunctional Requirements

The nonfunctional requirements identified are the following:

- Security: information about the system should be secure and safe.
- Maintenance: the system permits to be upgraded and modernized when it is necessary.
- Usability: the system can be accessed by the admin and the user without issues or difficulties.
- Performance: this system responds fast to users without any delay or complications.
- User Friendly: the complete system is developed in a standardized way which makes it user-friendly in terms of the interface and easiness concerning understanding its usage.

**Figure 4:**
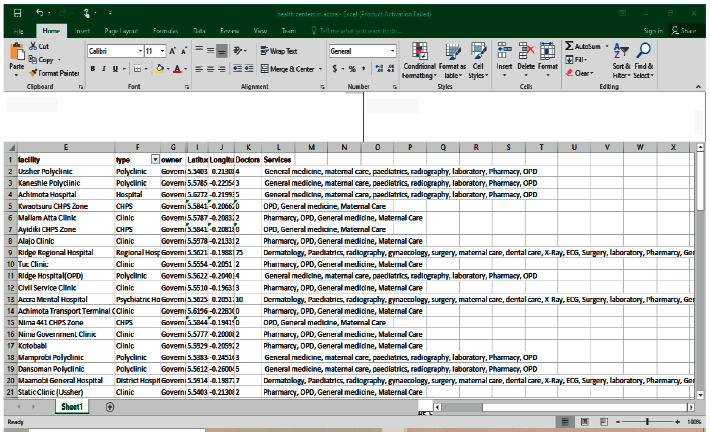
Organization of data attributes.

Figure 4 depicts an object-oriented database for geographic and attribute data. Data from ArcGIS Collector was entered into Microsoft Excel and saved as a CSV file in the same folder as the GPS data. The characteristics of the health facilities, as well as the GPS data associated with them, were then merged into a single database.

### 5.3. User Interface (UI) Design and Technologies

This section covers the ArcGIS screens' high-level technological design, which allows users to interact with specific constraints. Furthermore, this section describes the system's development process, from geographical data analysis through integration with ArcGIS online for further deployment. The geographic coordinates and characteristics were retrieved, processed, and put onto the ArcMap platform, as illustrated in Figure 4. The following are the procedures involved:

- Create a file geodatabase in ArcCatalog.
- Load the CSV data into the file geodatabase.
- Import the data into the ArcMap environment.
- Change the symbol to represent the points imported.

**Figure 5:**
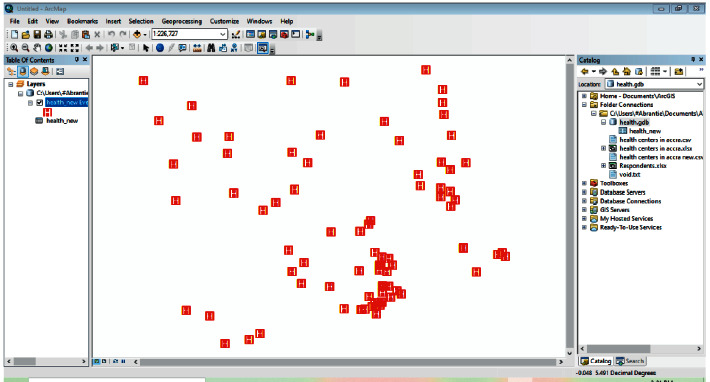
Coordinates and attributes on the ArcMap.

Figure 5 shows the feature classes, properties, and coordinates shown on ArcMap from a file geodatabase that has been modeled in the ArcCatalog. The feature services are provided as a service on the ArcGIS online platform with an active account from the ArcMap.

**Figure 6:**
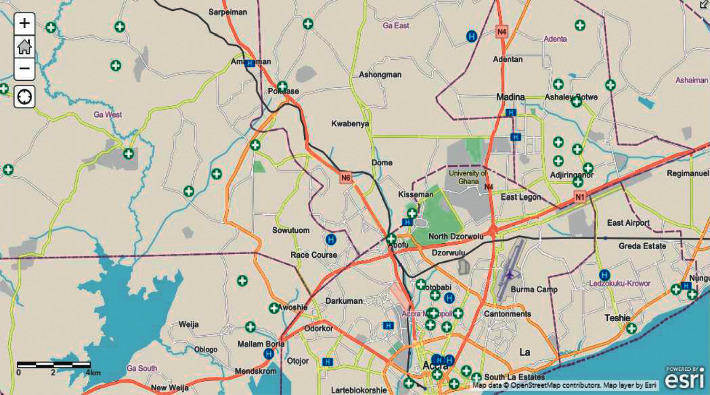
Map showing spatial analysis of all health centres.

Figure 6 is a representation of the *Geohealth* homepage, which displays all of the health centres and their many properties (facility name, district, subdistrict, owner, type, class, and services) for the user to engage with before going to the specific health centre they wish to visit.

**Figure 7:**
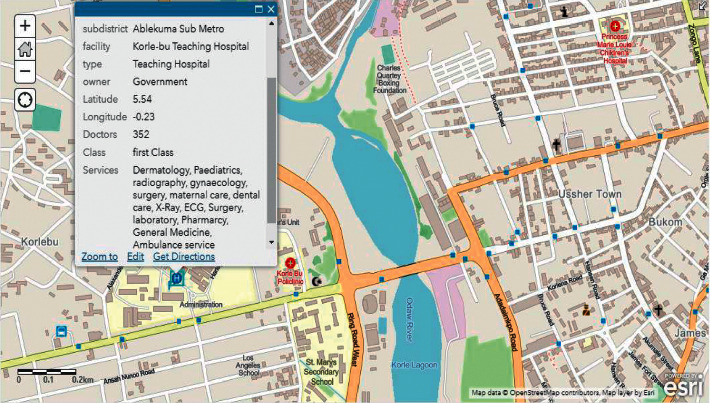
Spatial analysis of health centre location and services.

Figure 7 shows the user requested information about the health centre and the location.

**Figure 8:**
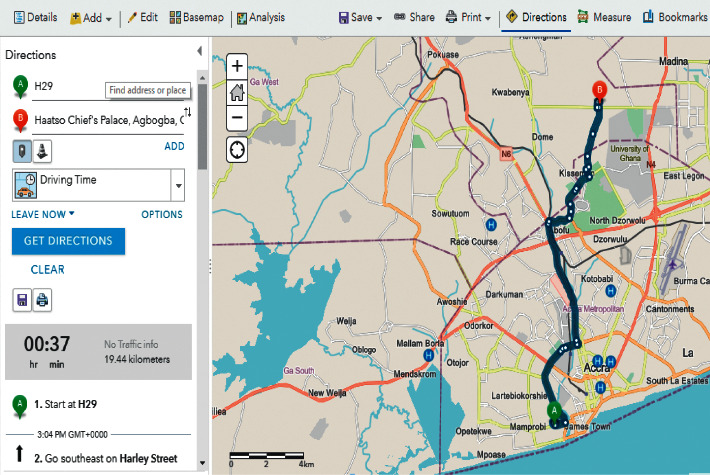
Spatial analysis of directions to the requested health centre.

Figure 8 depicts the destination's direction as well as other routes. This uses a voice command to lead the user from the take-off point to the destination. It may also be used if the user is driving and maybe handicapped. It also allows the user to select a traffic mode that decides whether or not there is vehicular traffic, as well as whether or not to depart right away or later (setting the time as well).

### 5.4. Evaluation of Geohealth

This section presents the performance and evaluation of our proposed *Geohealth system*. We utilize the data gathered initially in our GIS setup and illustrate the comparison of *Geohealth* as a computerized web-based map to Google Maps (evaluation benchmark). The evaluation parameters used involve location-based health centres in Accra and precise health services offered.

#### 5.4.1. Geohealth versus Google Maps

Several platforms could be used in this paper to integrate our data into a GIS, but we chose to collect our data using a different primary source to ensure the accuracy of the data collected, as well as to ensure that we collected all data that were not on Google Maps, which is a secondary data source. Data collected using the *Geohealth* system was passed through stages of quality control and assurance, which authenticated the data published on the web-based platform. Google Maps however relies on data inputted by users of the system (Google Maps), but, in the case of *Geohealth*, all data were collected at the source (health centres). We have highlighted the differences between *Geohealth* and Google Maps below.

*Geohealth* is a web-based map platform developed for public health centres in Accra and classified based on the various services they provide. Other data collected from primary sources included the number of medical personnel, whether they are National Health Insurance Scheme (NHIS) accredited, and accurate contact information using *Esri products* such as *ArcMap*, *ArcCatalog*, *ArcScene*, *ArcGIS Online*, and *ArcGIS Collector*.

The gathered data were initially modeled in ArcGIS Diagrammer before being exported to ArcMap for feature class and domain definition. As a result, the data obtained will be more efficient and accurate. Furthermore, *Geohealth* is a web-based map dedicated solely to Accra's public health centres, which is accessible online or through mobile, ensuring the data's integrity.

On the other hand, Google Maps is a consumer application and web mapping platform developed by Google. Satellite images, aerial photos, street maps, 360° interactive panoramic views of streets, real-time traffic conditions, and route planning for walking, driving, flying, and taking public transportation are all available. Google Maps is a web-based service that provides extensive information on geographic areas and locations all over the world. Aerial and satellite views of numerous locations are available on Google Maps in addition to traditional road maps. Google Maps provide street views based on pictures obtained from automobiles in various cities.

#### 5.4.2. Discussion Evaluation Results

**Figure 9:**
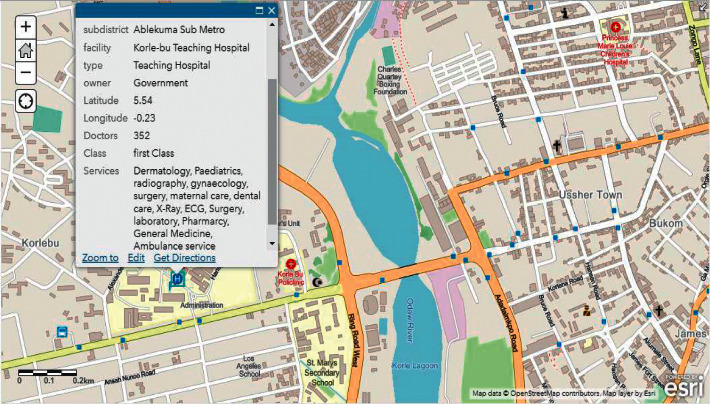
Geohealth information on KBTH.

**Figure 10:**
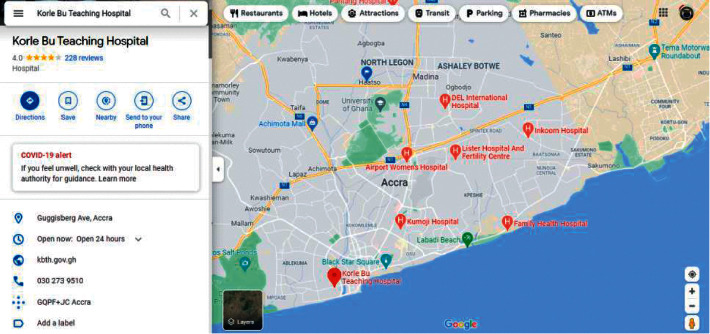
Google Maps information on KBTH.

**Figure 11:**
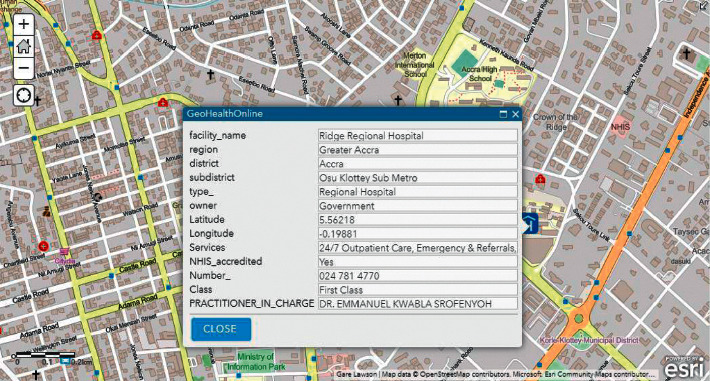
Geohealth information on RRH.

**Figure 12:**
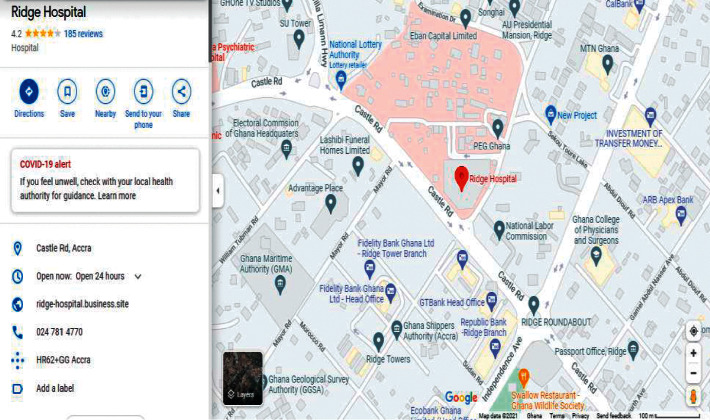
Google Maps information on RRH.

During our evaluation, we employed two major health centres in Accra as benchmarks. These health centres include Korle Bu Teaching Hospital (KBTH) and Ridge Regional Hospital (RRH). It must be noted that, due to the required length of our paper, evaluation results for the other public health centres can be found in our Supplementary Materials. Figures 9to 12 illustrate the system performance of *Geohealth* and Google Maps in terms of our aforementioned evaluation parameters using the benchmark health centres above. In summary, as shown in Table 8, in terms of the location, ownership status, classification status, and provision of services, G*eohealth* outperforms Google Maps in both health centres (KBTH and RRH).

## 6. Concluding Remarks

In this paper, we developed a computerized web-based map called *Geohealth* for categorizing public health clinics in Accra using GIS technology. Globally, the fast expansion of ICT in healthcare delivery has enabled the utilization of technology in healthcare services. With reference to our analysis and results in this paper, the successful implementation and use of *Geohealth* for public health facilities in Accra are highly applicable and appropriate. Full embracement of *Geohealth* will guarantee several benefits, such as saving of time and effort by directing users securely and effortlessly to the health facility which provides the exact services required.

We used the UTAUT paradigm to design a quantitative method (questionnaire via online Google Forms). 260 complete responses were received for effective data analysis. Quantitative results in this paper show that a majority of the participants in the research are eager to accept *Geohealth* as a tool for obtaining information and data on Accra's health centres. Furthermore, we evaluated the performance of *Geohealth* in comparison to Google Maps as a benchmark. Evaluation results show that, using both KBTH and RRH, *Geohealth* performs better than Google Maps in terms of precise location, ownership status, classification status, and provision of services, which enables users to make appropriate and well-informed decisions.

In summary, based on the analysis and evaluation of the proposed system, it can be safely stated that the proposed system is an efficient, useful, and dependable system that benefits users as well as the nation as a whole in the sphere of national development.

Projecting the geographical distribution of the data obtained on a map is the best process for illustration and analysis. GIS is one of the best tools for dealing with such information. As part of our ongoing research progress and future work, disease search or symptom detection will be integrated into *Geohealth*. Data collection will need to be repeated to guarantee that the improved system identifies disease outbreak symptoms on the platform and then recommends a health facility that can respond to the situation [23].

## Figures and Tables

**Table 1:** Profile of respondents.

No.	Variable	Category	Respondents		
			*N*		%
1	Gender	Male	160		61.5
		Female	100		38.5

		Total	260		100.0

2	Age group	15–24 years	44		16.9
		25–34 years	113		43.5
		35–44 years	38		14.6
		45–54 years	64		24.6
		55 and above	1		0.4

3	Educational level	Total	260		100.0
		SSCE	9		3.5
		WASSCE	92		35.4
		BSc	77		29.6
		MSc	56		21.5
		Others	26		10

		Total	260		100.0

**Table 2:** Technology acceptance of respondents.

Questions	TN	*M*	SE	SD	*V*
Did you find it easy to locate the health centre you intended to visit?	260	1.77	0.03	0.42	0.18
Did they offer the services you required?	260	1.73	0.03	0.44	0.20
Did you get any bed allocation immediately during admission?	260	1.57	0.03	0.50	0.25
Did they have the facilities to administer to your situation?	260	1.39	0.03	0.49	0.24
Have you ever heard of the term *Geohealth*?	260	1.27	0.03	0.45	0.20
Have you been turned away by any health centre because they don't offer the services you required?	260	1.18	0.02	0.39	0.15
Have you been admitted into a health centre for any illness?	260	1.10	0.02	0.31	0.09
Would you accept *Geohealth* as a method of accessing information about public health centres?	260	1.03	0.01	0.16	0.03
Do you use smartphone?	260	1.00	0.00	0.06	0.00

**Table 3:** TPE of respondents.

Questions	TN	*M*	SE	SD	*V*
I will find *Geohealth* useful and efficient in access to health centres?	260	1.10	0.02	0.31	0.09
Using *Geohealth* would enable me to access health care services faster and easily?	260	1.10	0.02	0.30	0.09
Using *Geohealth* will enable me to manage time efficiently, since I can check health centres closer to me and also their services before setting out?	260	1.09	0.02	0.29	0.08

**Table 4:** TEE of respondents.

Questions	TN	*M*	SE	SD	*V*
I will find *Geohealth* easy to use?	260	1.11	0.02	0.31	0.10
It is easy for me to become skillful at using *Geohealth*?	260	1.10	0.02	0.31	0.09
My interaction with *Geohealth* would be clear and understandable towards my wellbeing?	260	1.10	0.02	0.31	0.09
Learning to operate *Geohealth* will be easy for me?	260	1.10	0.02	0.30	0.09

**Table 5:** SI of respondents.

Questions	TN	M	SE	SD	V
People who influence my behavior would think that I should use it?	260	1.14	0.02	0.35	0.12
People who are important to me would think that using *Geohealth* is good for me?	260	1.12	0.02	0.32	0.10

**Table 6:** FC of respondents.

Questions	TN	*M*	SE	SD	*V*
*Geohealth* is not accessible on the browser I use?	260	1.66	0.03	0.48	0.23
Help is available for me should there be any difficulties in using *Geohealth*?	260	1.16	0.02	0.37	0.13
I have the resources necessary to use *Geohealth*?	260	1.15	0.02	0.35	0.13
I have the knowledge necessary to use *Geohealth*?	260	1.14	0.02	0.35	0.12

**Table 7:** BI of respondents.

Questions	TN	*M*	SE	SD	*V*
I intend to recommend *Geohealth* to friends and family in the future?	260	1.10	0.02	0.31	0.09
I predict that I would use *Geohealth* in the future?	260	1.10	0.02	0.31	0.09
I intend to use *Geohealth* in the future?	260	1.10	0.02	0.31	0.09
I plan to use *Geohealth* in the future?	260	1.09	0.02	0.29	0.08

**Table 8:** Geohealth versus Google Maps (KBTH).

Geohealth	Google Maps
Figures 9 and 11 show further information about the location, such as the subdistrict, district, and region of KBTH and RRH, respectively.	In Figures 10 and 12, there is no detailed information about the queried location of KBTH and RRH as compared to *Geohealth* in Figures 9 and 11.
Figures 9 and 11 clearly illustrate the ownership status of KBTH and RRH, respectively. Concerning whether they are owned by the government or privately owned, this will help guide the user to make informed decisions about which health centre to visit.	Figures 10 and 12 lack information on the respective ownership statuses of KBTH and RRH, that is, whether they are government-owned or privately owned facilities.
One of the key components of *Geohealth* is the classification status of the health centre. Figures 9 and 11 illustrate that KBTH and RRH are first-class health centres because they meet all the criteria required to qualify for that category.	There is no information or data on the class of health centre of both KBTH and RRH, respectively, in Google Maps (Figures 10 and 12).
In Figures 9 and 11, data from KBTH and RRH include a clear depiction of the services provided by the health centre. This provides the end-user with accurate and informed information about the health facility before their visit.	In Figures 10 and 12, there is no information on the KBTH and RRH services in respective Google Maps.

## Data Availability

The data used to support the findings of this study are included within the article. The authors utilized and interconnected various components of dataset and feature classes, namely, GeohealthOnline.csv, GeoHealthOnline.mxd, GeoHealth.diagram, and GeohealthOnline.xml, which is available on Google Drive at https://drive.google.com/drive/folders/1iC17q_tbGlvKzKZ9nMVDeoO2RVSdGipu?usp=sharing, and the responses from respondents on Google Form are available at https://forms.gle/44rDwLGu4L5xuzwB8.

## Supplementary Materials

Supplementary Materials The supplementary material of this paper elaborates on the dataset which was utilized in the development of *Geohealth*. A dataset description of the Pictorial-Representation-of-the-Various-Components-of-the-*Geohealth*-System, which can be accessed through the links below, is appropriately provided in the supplementary material. Additionally, in relation to evaluation performance, the supplementary material also contains figurative evaluation results of other public health centres in Accra apart from KBTH and RRH within the article. Furthermore, the supplementary material also contains a short video concerning the development process of *Geohealth*.
