# Supplementary material for: Classification of Public Health Centres in Accra through a Web-Based Portal Integrated with Geographical Information System (GIS)
Source: J Healthc Eng. 2021 Dec 3;2021:4178161. doi: 10.1155/2021/4178161 (PMC8664506; doi:10.1155/2021/4178161)
Supplement: Supplementary Materials — The supplementary material of this paper elaborates on the dataset which was utilized in the development of Geohealth. A dataset description of the Pictorial-Representation-of-the-Various-Components-of-the-Geohealth-System, which can be accessed through the links below, is appropriately provided in the supplementary material. Additionally, in relation to evaluation performance, the supplementary material also contains figurative evaluation results of other public health centres in Accra apart from KBTH and RRH within the article. Furthermore, the supplementary material also contains a short video concerning the development process of Geohealth. [file 4178161.f1.pdf]

AUTHORS/RESEARCHERS:

Nana Yaw Asabere[1], Gare Lawson[1,2], Godwin Badu-Marfo[2], Lydia Kwofie[1], Daniel Opoku Mensah[2] and Reginald Lartey[1]

[1] Department of Computer Science, Faculty of Applied Sciences, Accra Technical University (ATU), Accra, Ghana.
[2] DexAfrica Limited, Accra, Ghana.

Correspondence E-Mail: yawasabere2005@yahoo.com; yawasbere2011@gmail.com
RESEARCH ARTICLE: Classification of Public Health Centres in Accra Through a Web-Based Portal Integrated with Geographical Information System (GIS)
DATASET: Pictorial-Representation-of-the-Various-Components-of-the-GEOHEALTH-System
We utilized and interconnected various components of data set and feature classes namely: GeohealthOnline.csv, GeoHealthOnline.mxd, GeoHealth.diagram, GeohealthOnline.xml, which is available in Google Drive at:

https://drive.google.com/drive/folders/1iC17q_tbGlvKzKZ9nMVDeoO2RVSdGipu?usp=sharing

and the

Responses from respondents in Google form available at https://forms.gle/44rDwLGu4L5xuzwB8

In this section of the study some noteworthy points of importance which tends to differentiate the Geohealth system from the traditional google map of accessing information on public health centers in Accra. The first is that the method of data gathering for health centers employing the Geohealth system differs from that of the Google Maps data collecting procedure. A complicated link between the various entities, data collection, classification, data processing technique, and data components Second, there are both direct and indirect variables that influence the data gathered by different systems. The factors affecting direct providers are shown in the figures below.

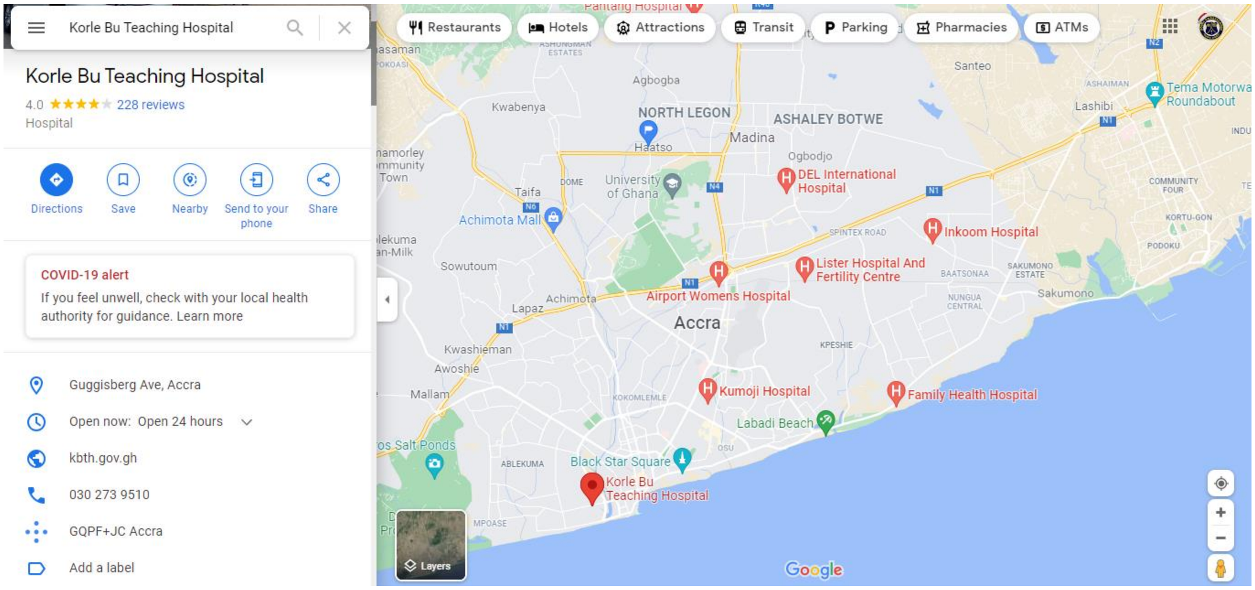

Figure 1: Google Map information on Korle-Bu Teaching Hospital

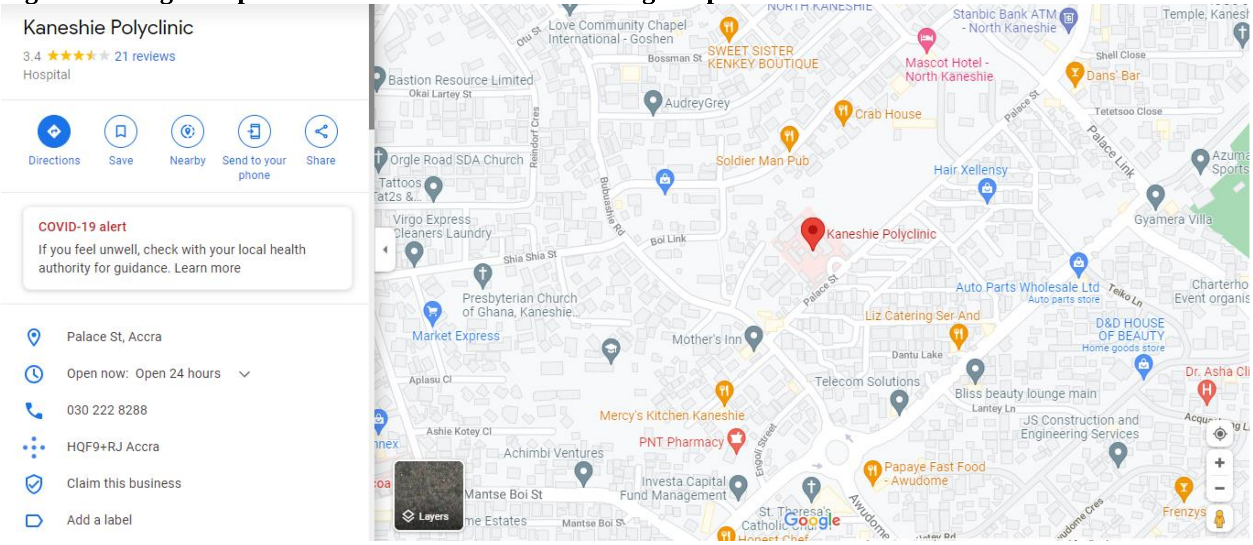

Figure 2: Google map information Kaneshie Polyclinic

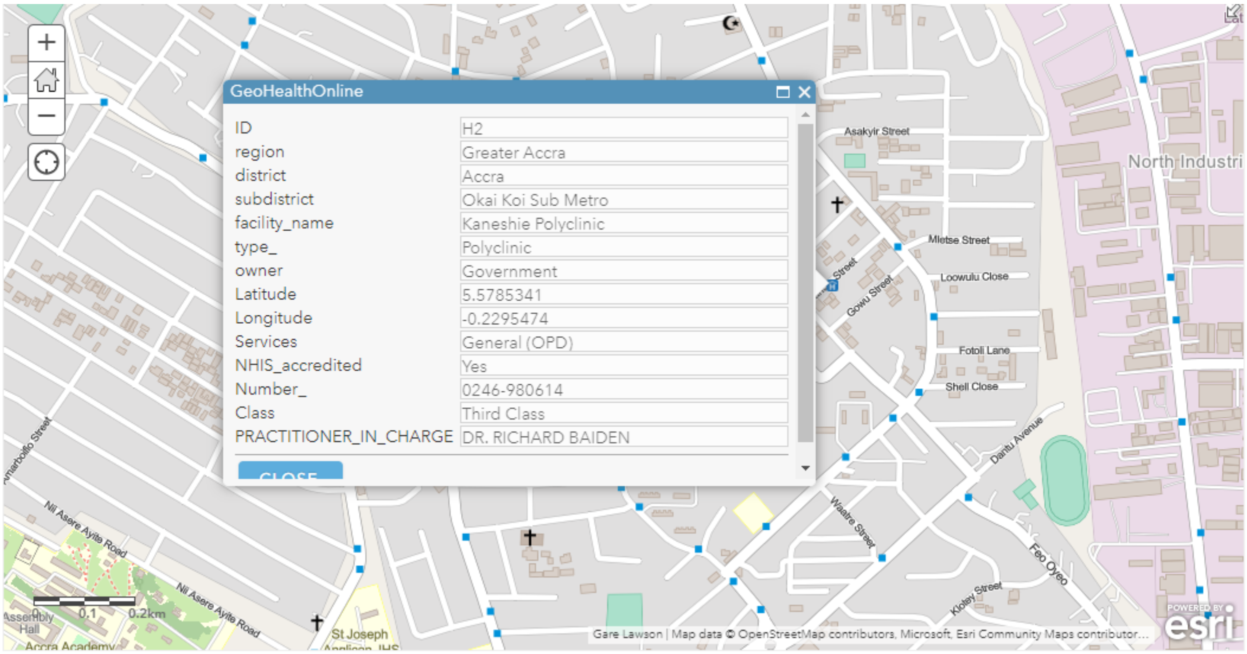

Figure 3: Geohealth information on Kaneshie Polyclinic

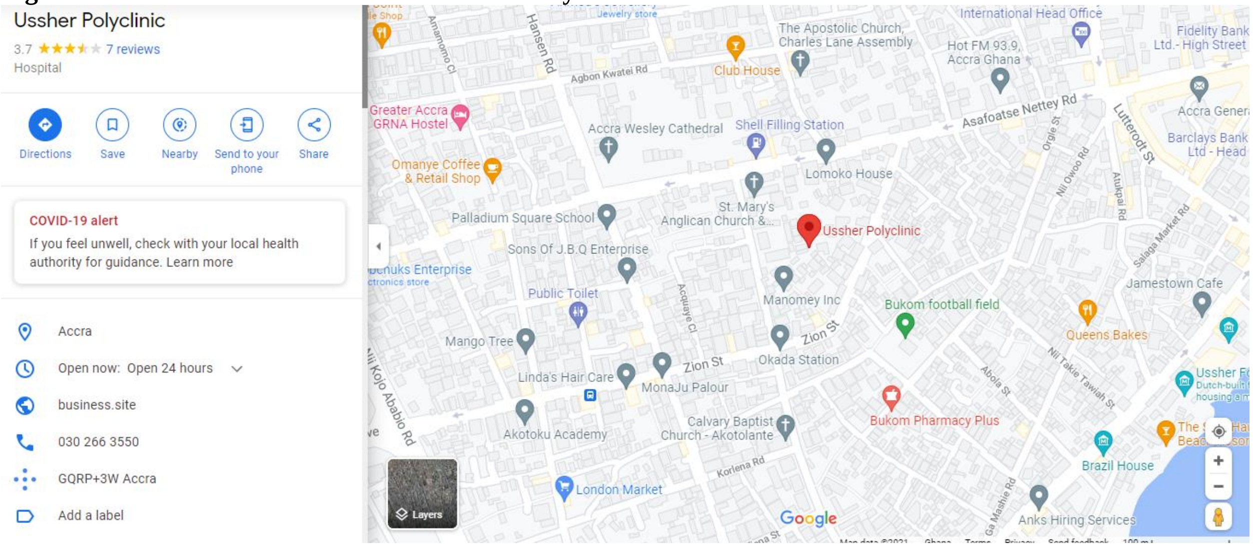

Figure 4: Google map information on Ussher Polyclinic

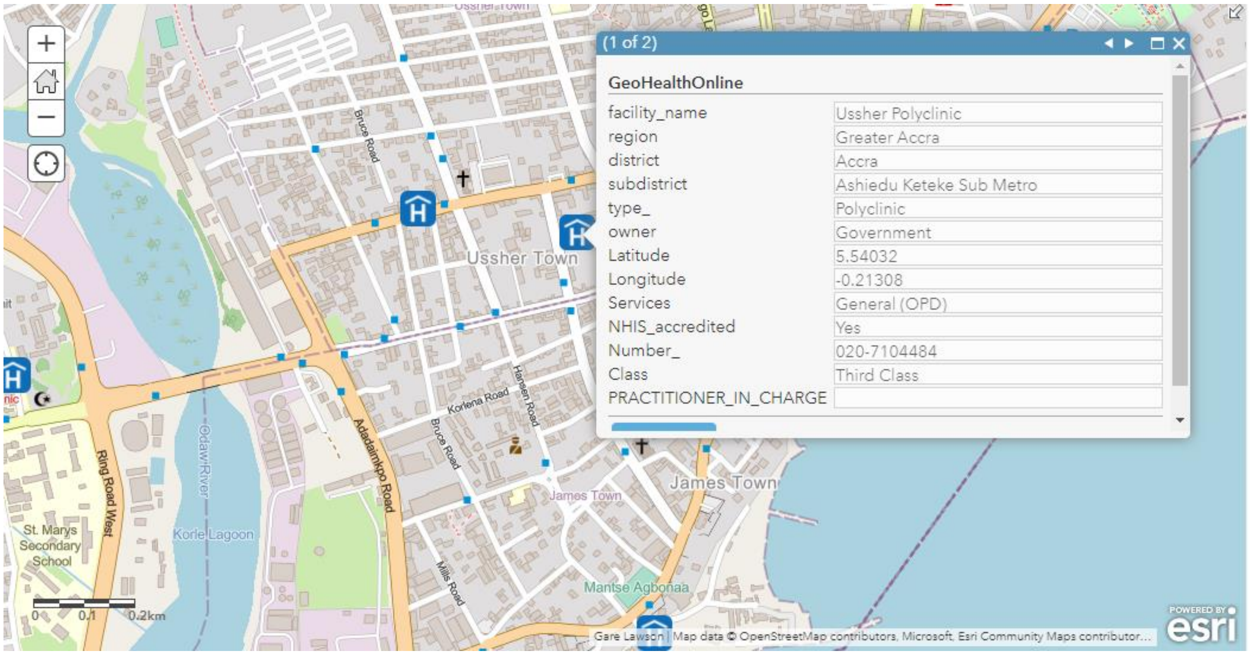

Figure 5: Geohealth Information on Ussher Polyclinic

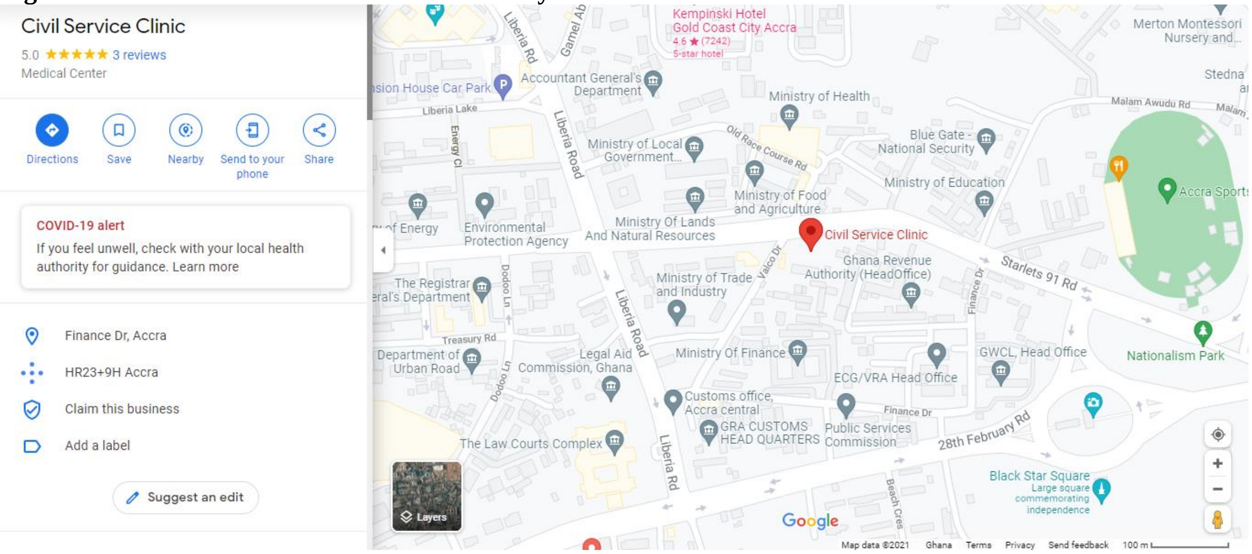

Figure 6: Google map information on Civil Service Clinic

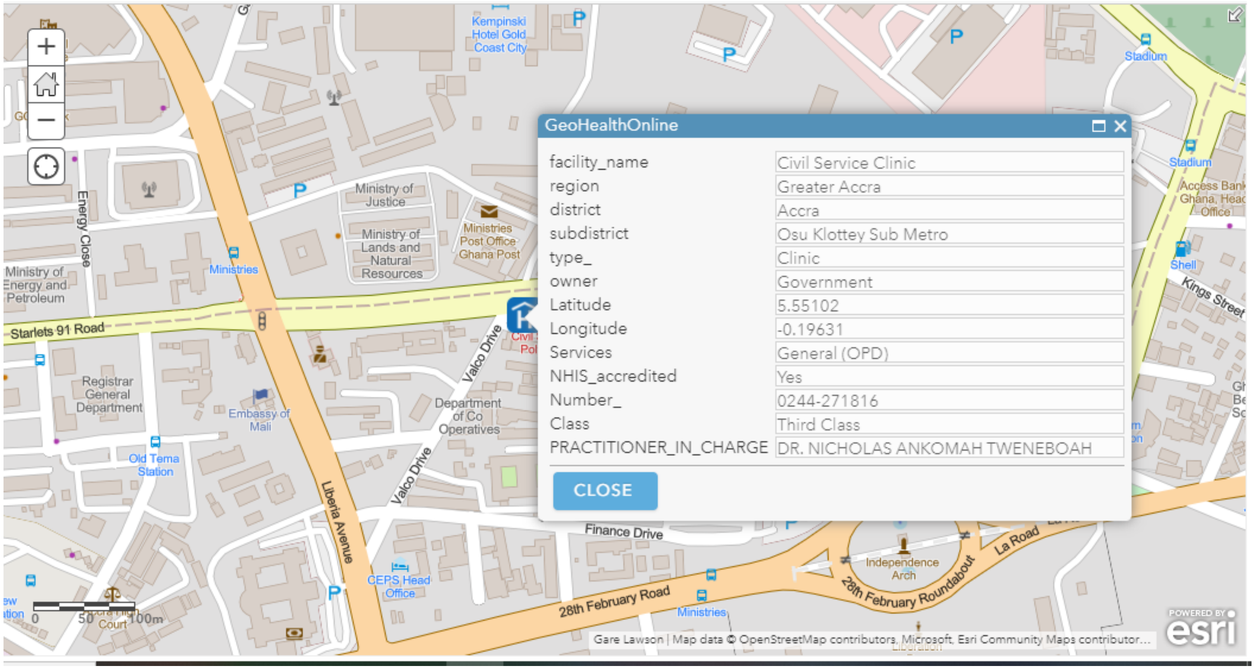

Figure 7: Geohealth information on Civil Service Clinic

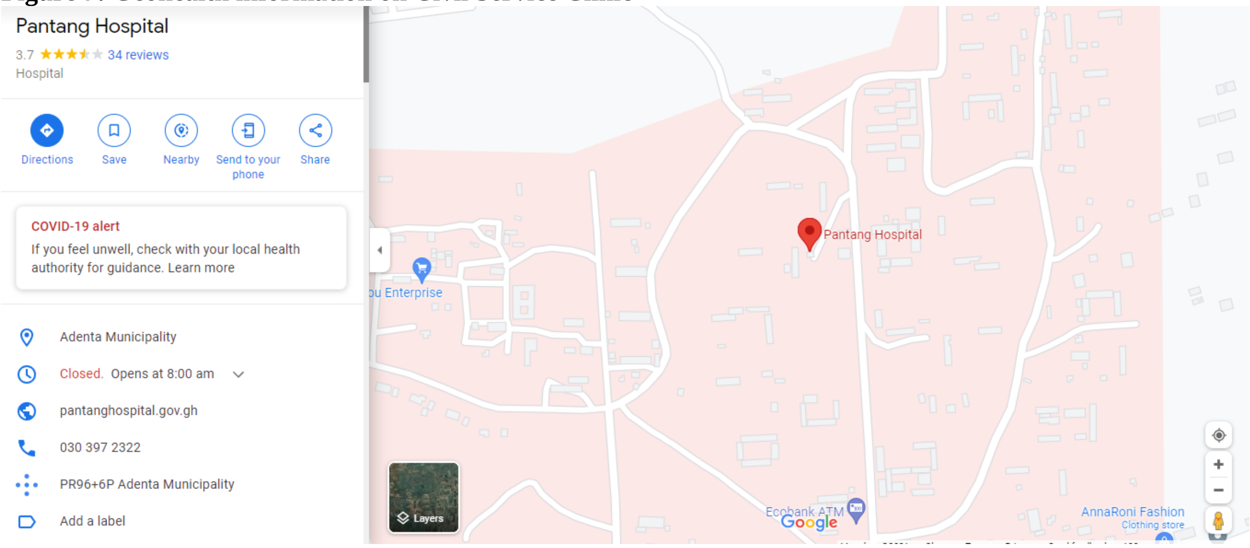

Figure 8: Google Map Information on Pantang Hospital

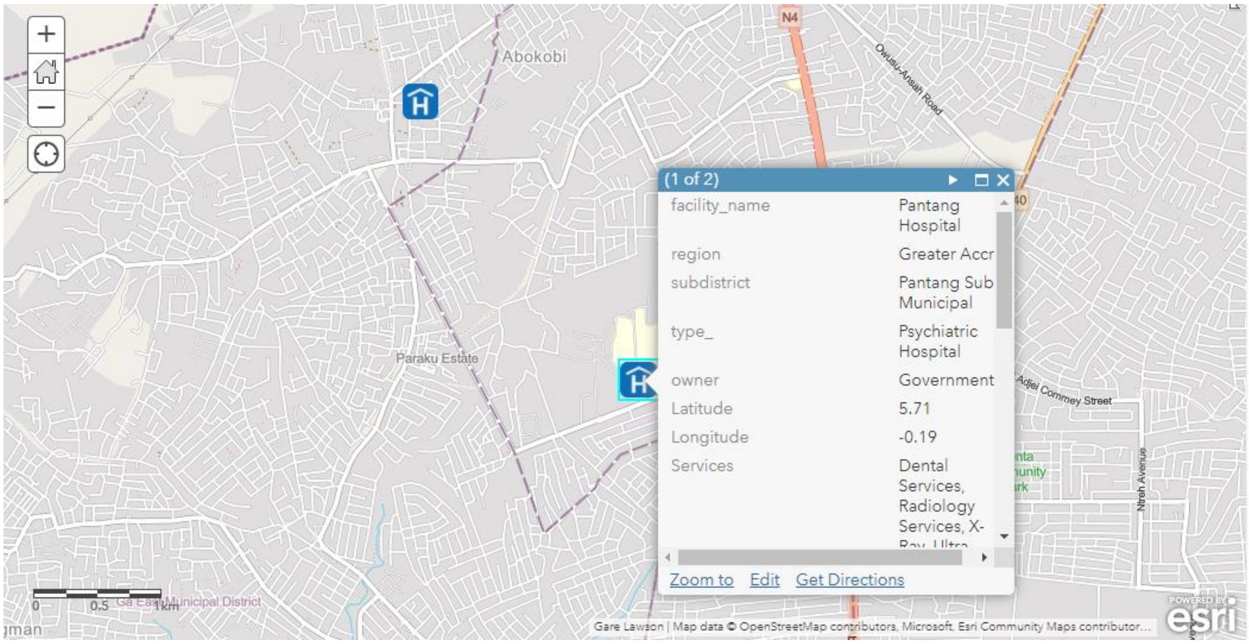

Figure 9: Geohealth Information on Pantang Hospital

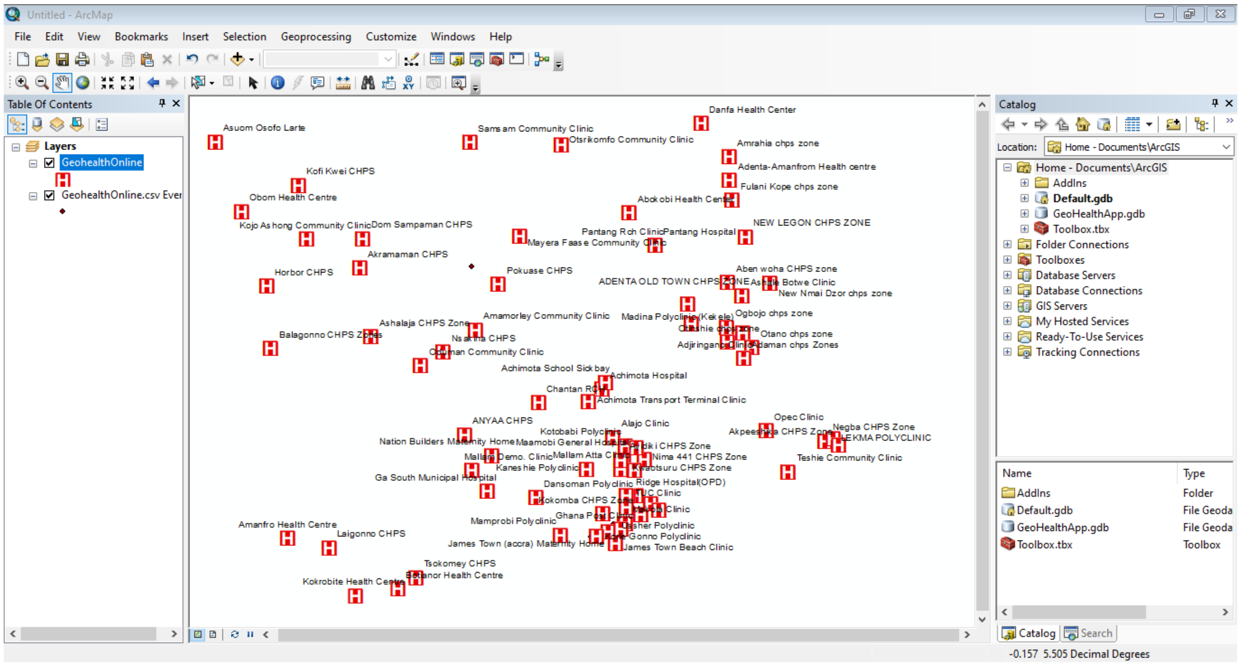

Figure 10: Feature Classes of the Health Centers imported onto ArcMap

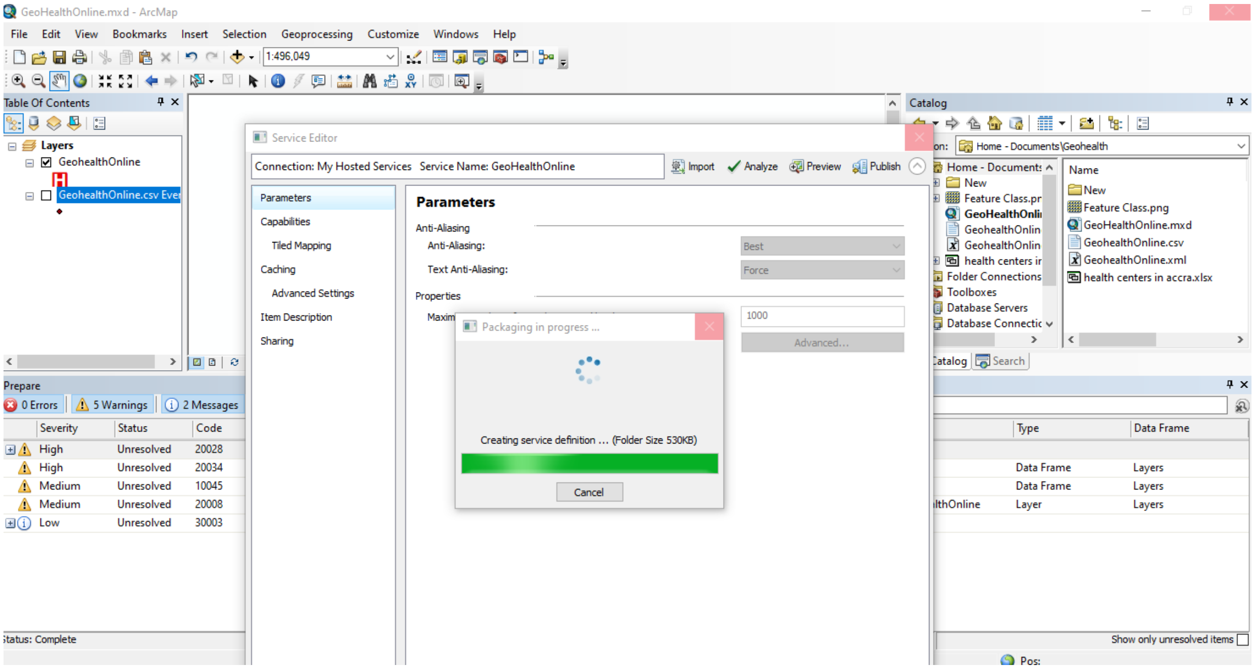

Figure 11: Published Service of the Health Centers onto ArcGIS Online

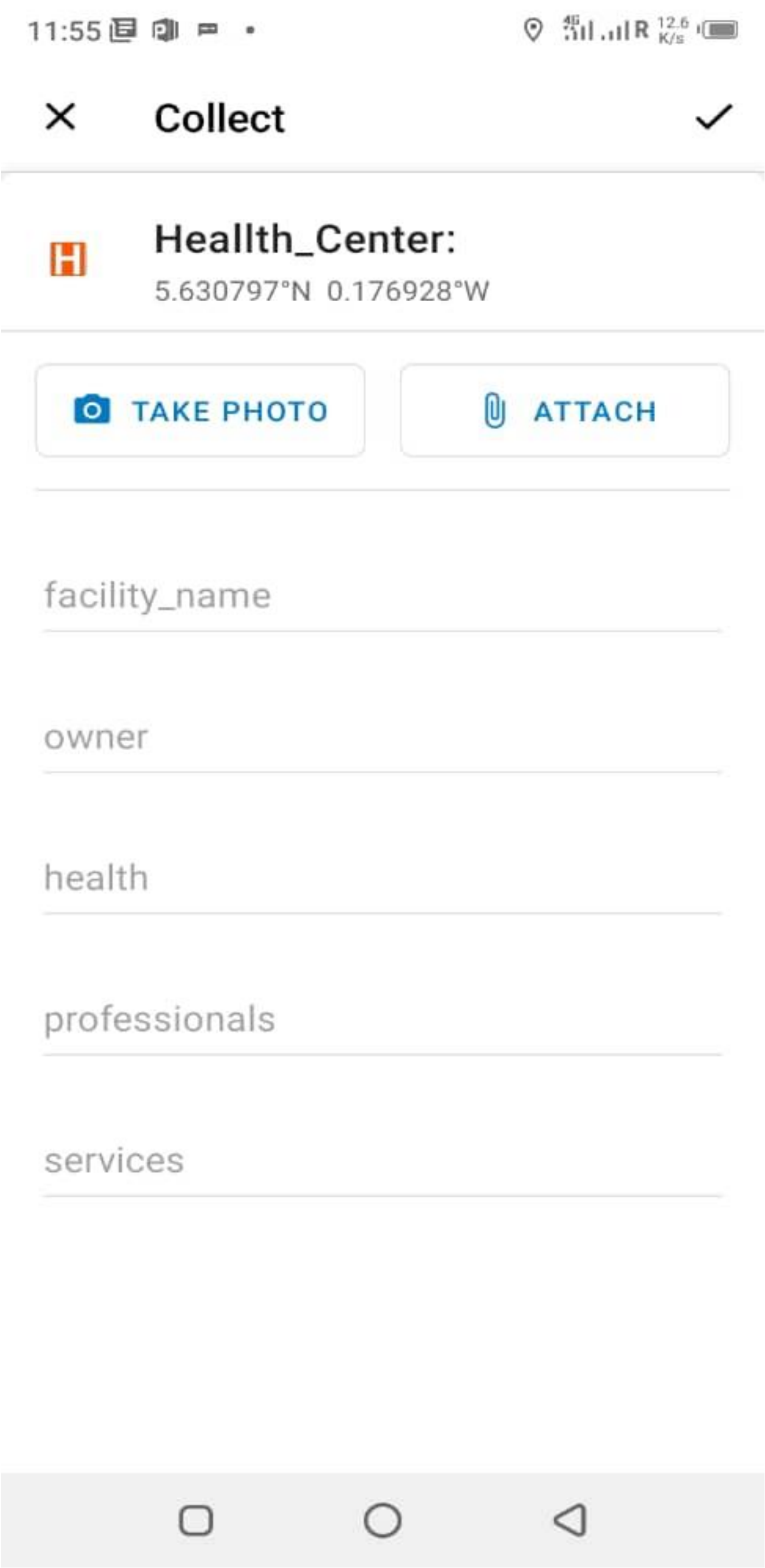

Figure 12: ArcGIS Mobile Collector App for Geohealth

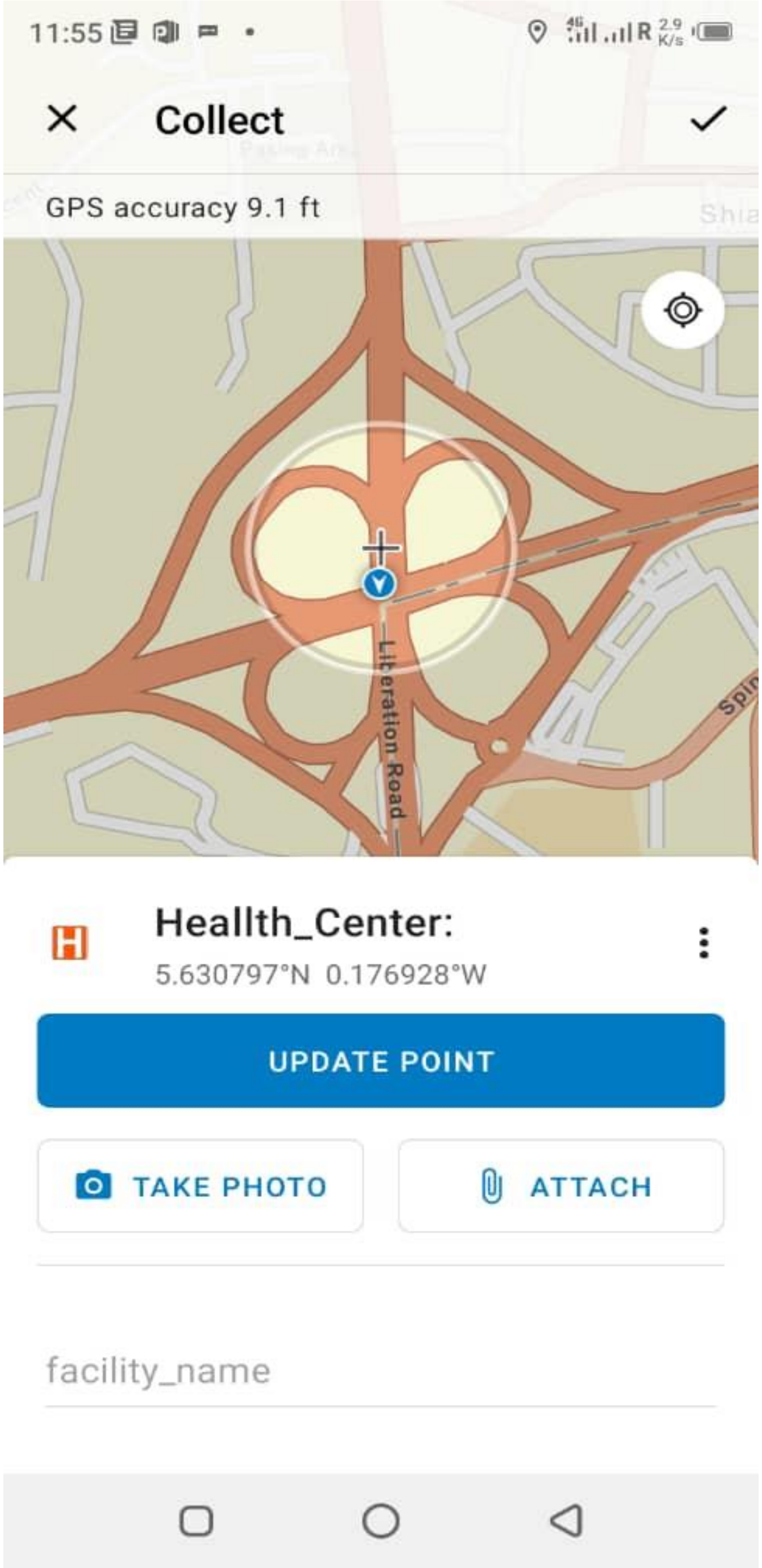

Figure 13: ArcGIS Mobile Collector App depicting how to pick Geolocation (Latitude and Longitude)

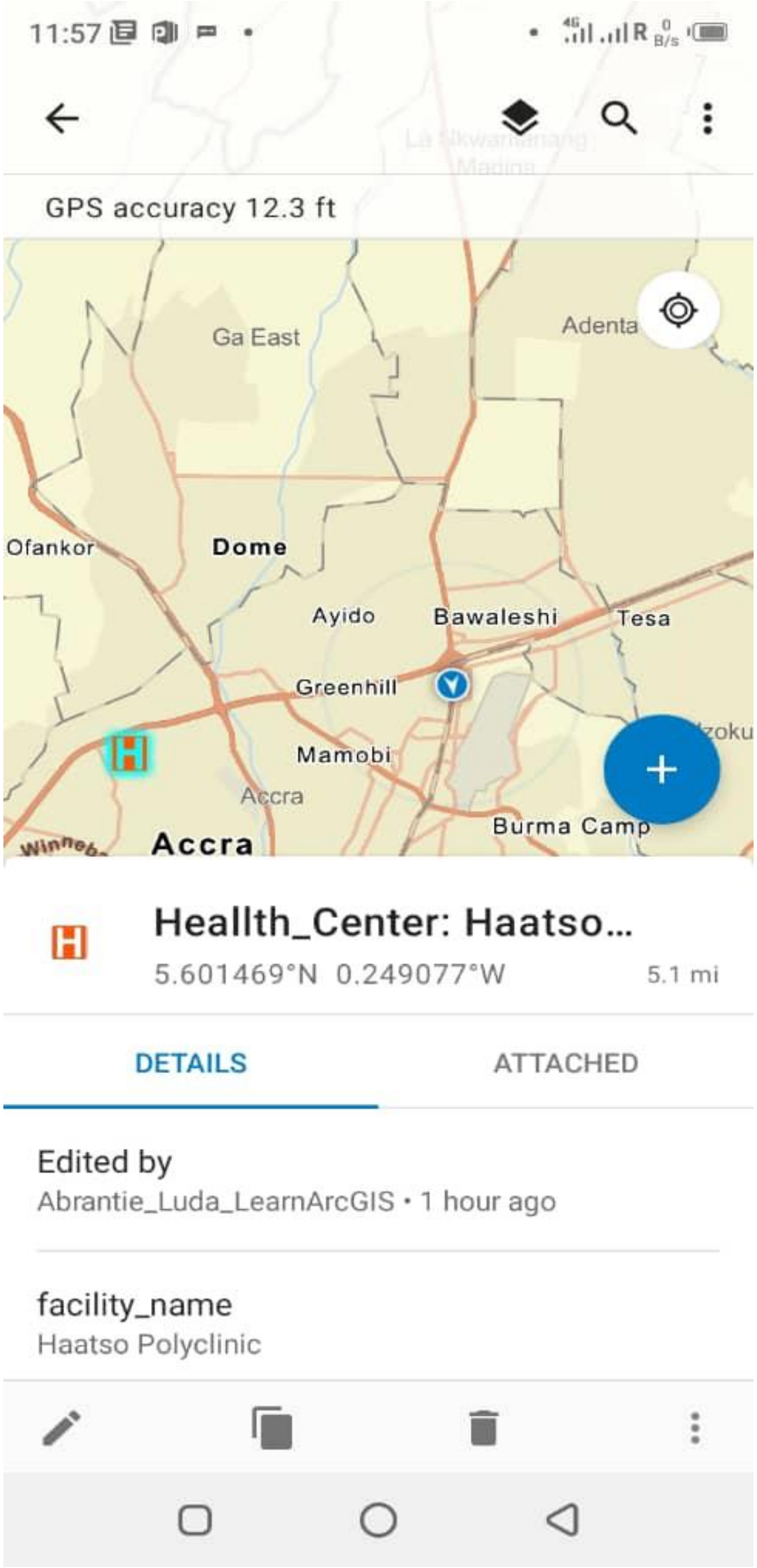

Figure 14: Sample Data of an Health Center Collected Using the ArcGIS Mobile Collector

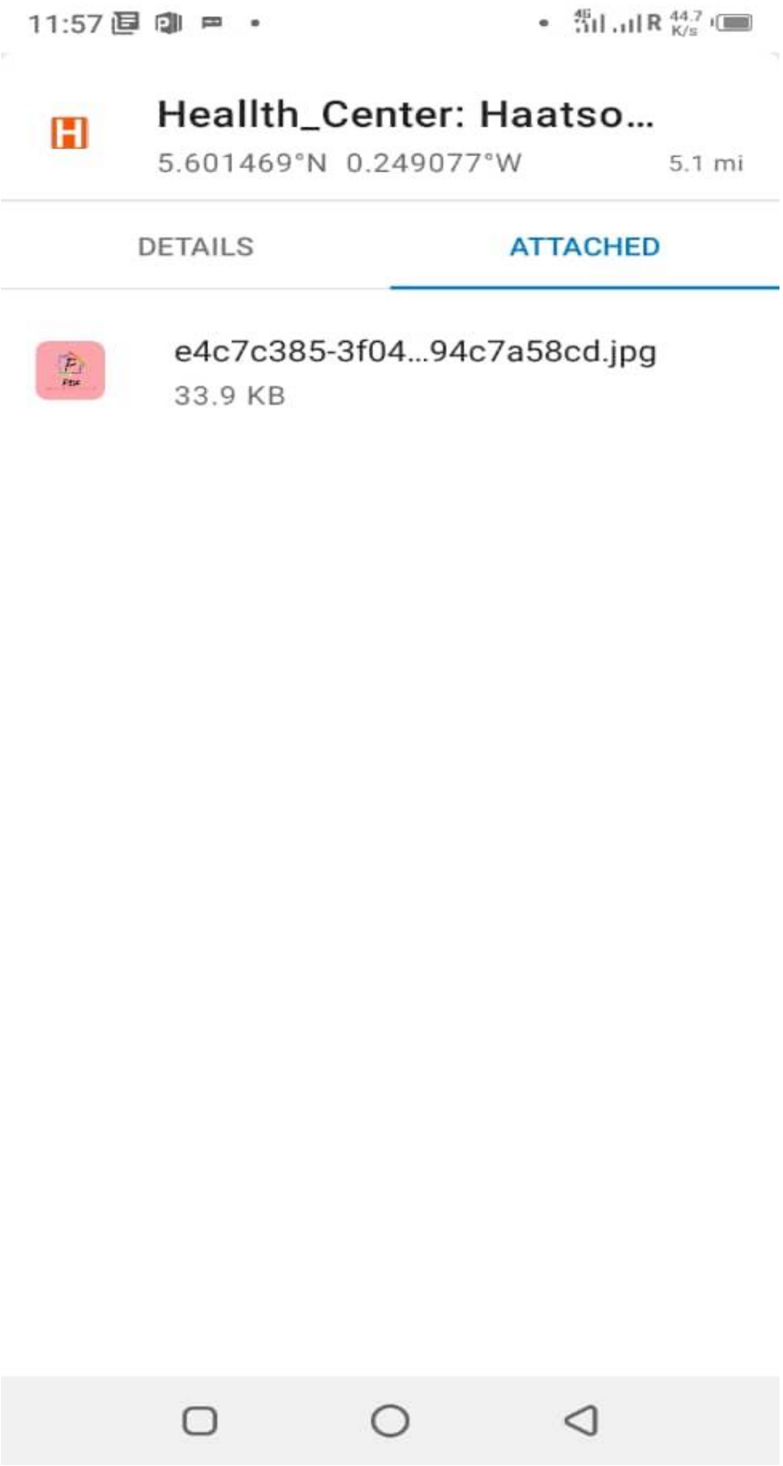

Figure 15: Sample of Image of the sampled Health Center Collected

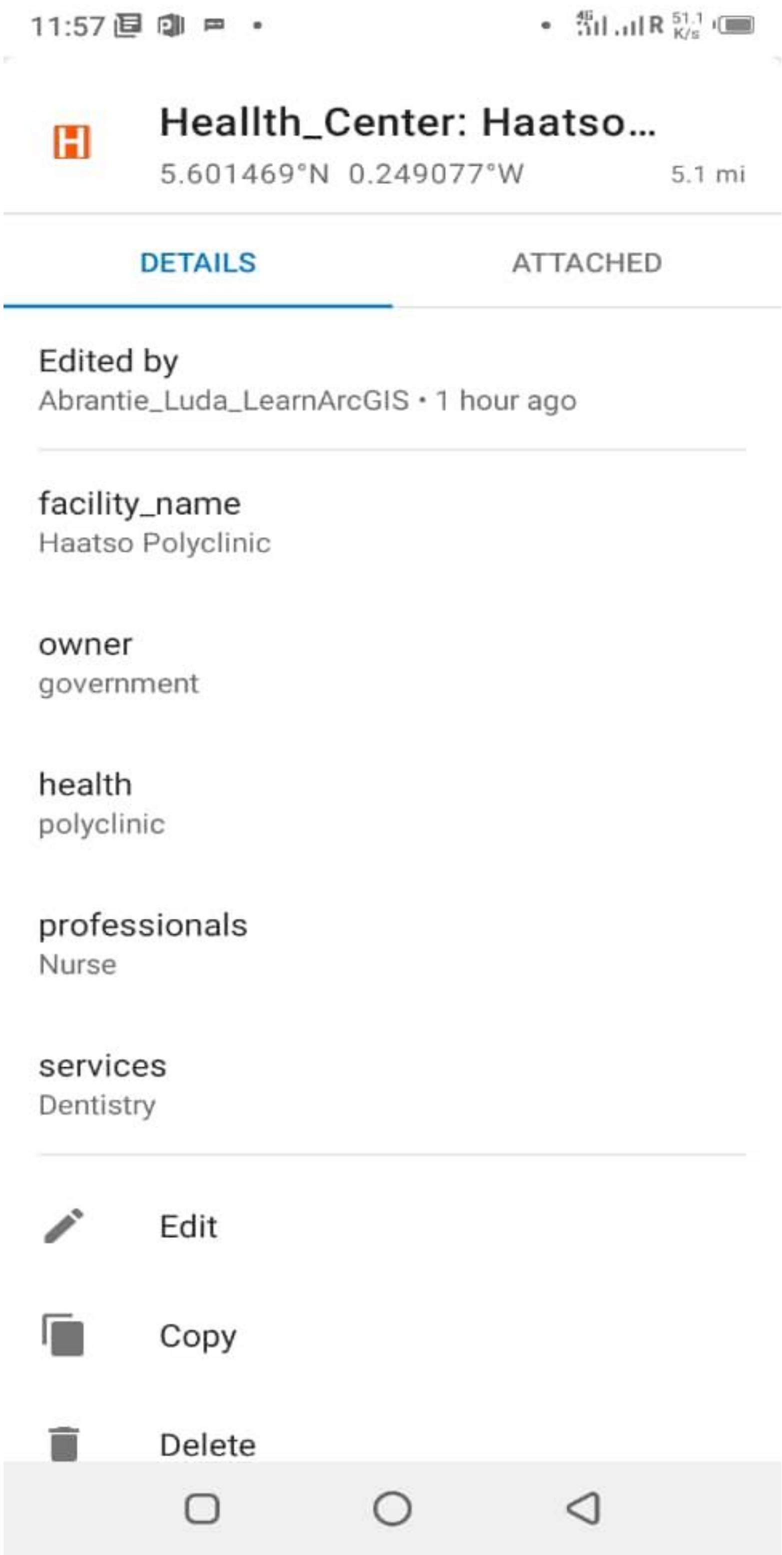

Figure 16: Visual Presentation of the Sample Data of Health Center Collected.

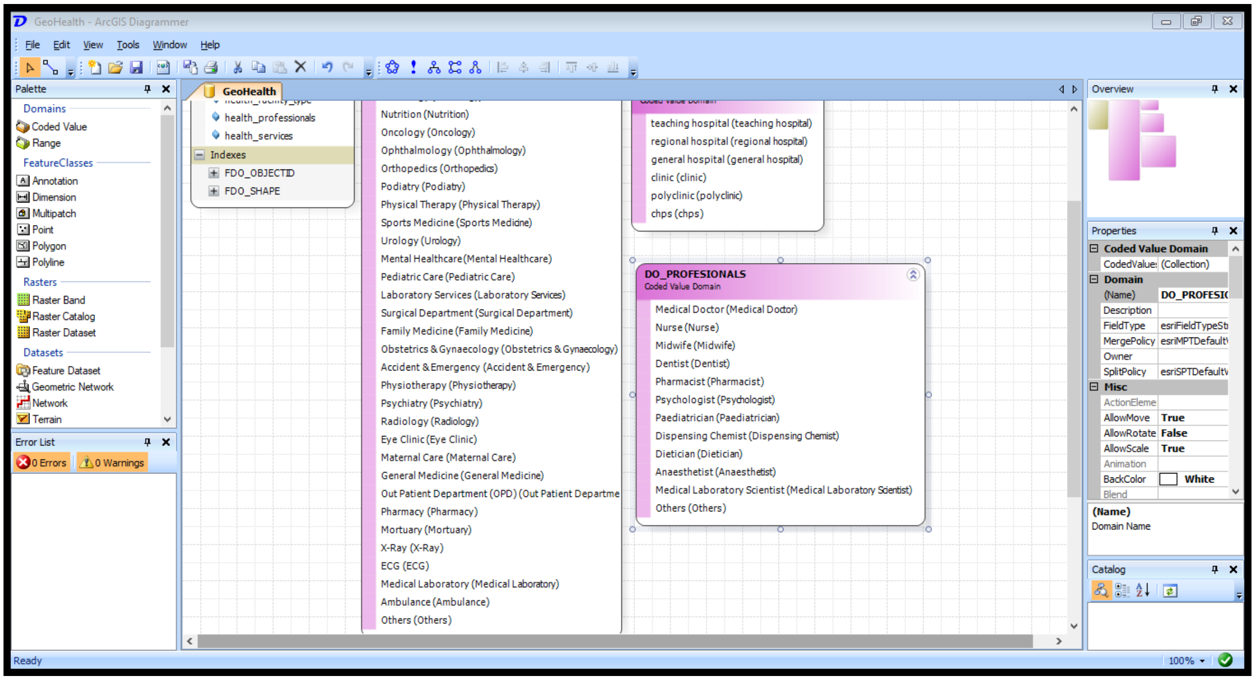

Figure 17: Geohealth ArcGIS Diagrammer XML File

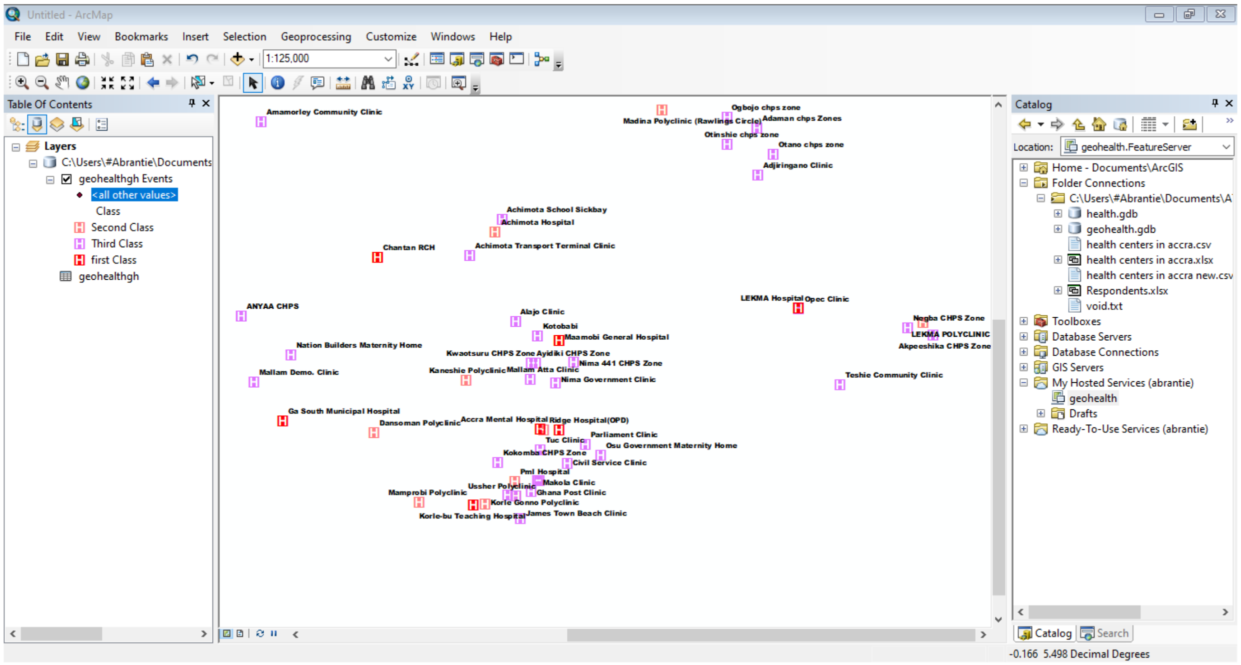

Figure 18: Geohealth File Geodatabase With all Attributes

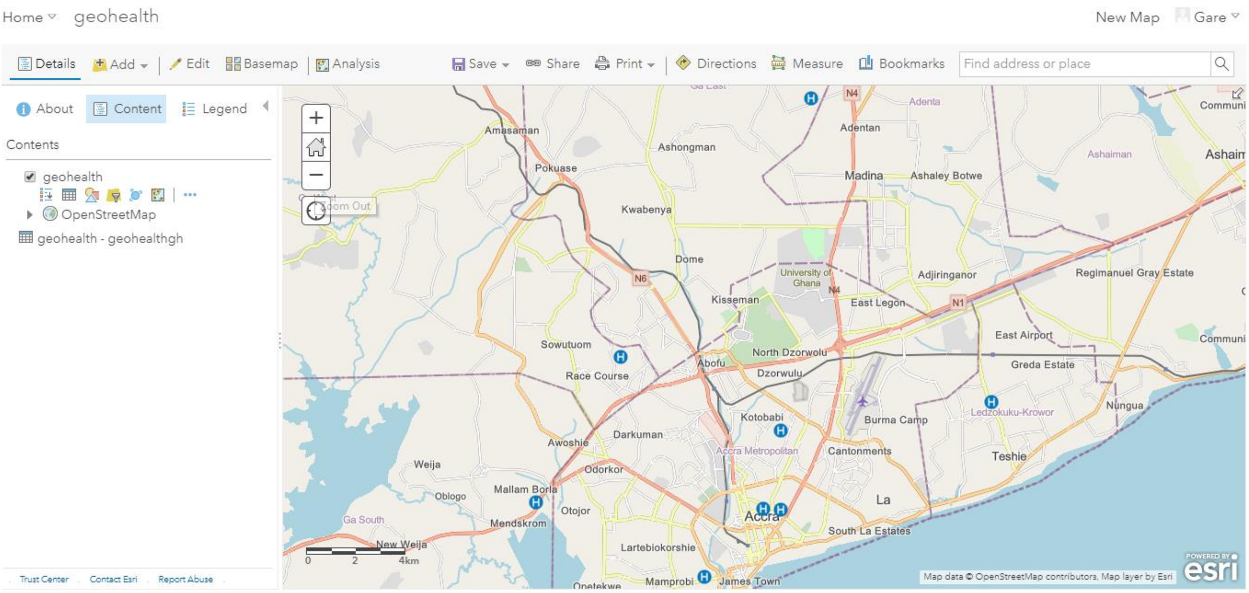

Figure 19: Spatial Analysis of First-Class Health Centers

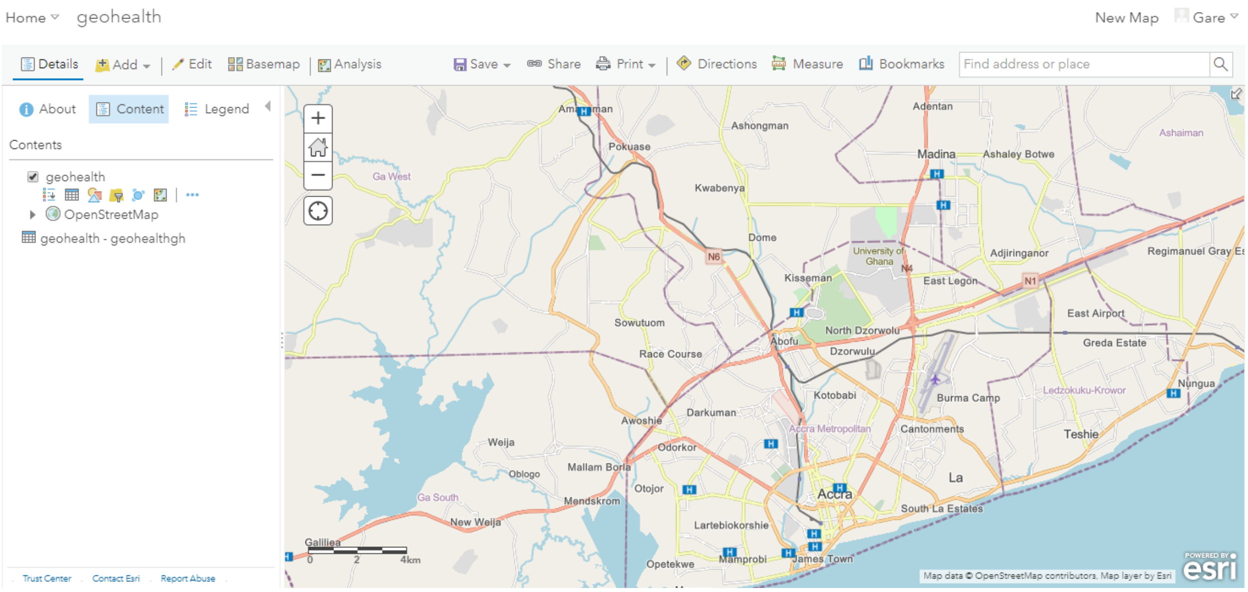

Figure 20: Spatial Analysis of Second-Class Health Centers

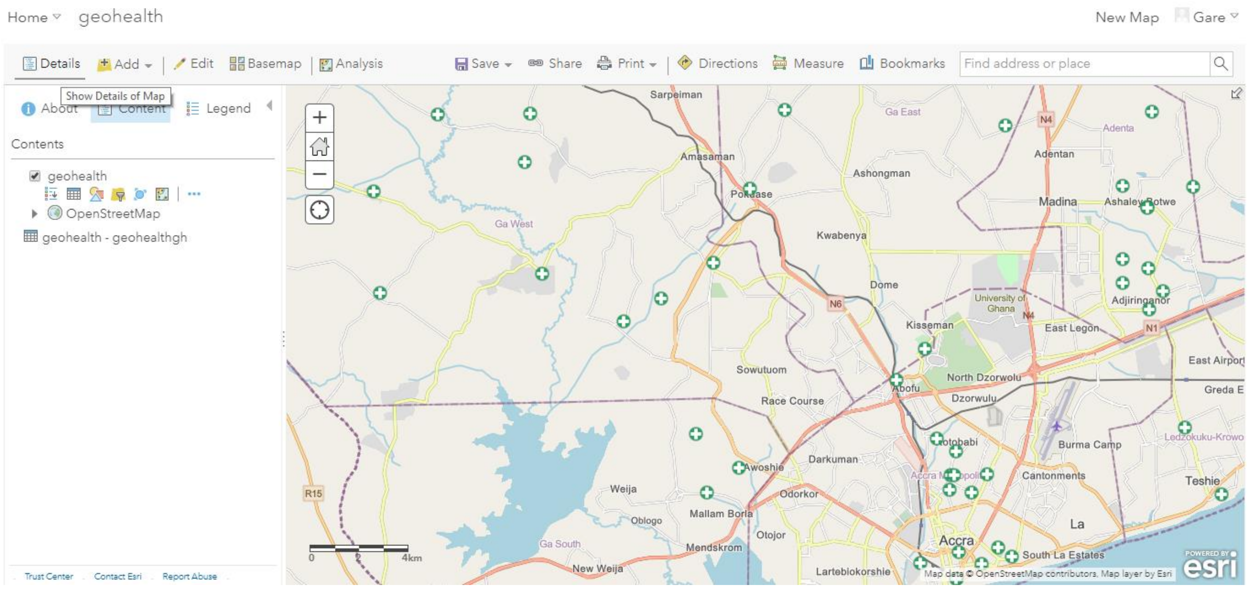

Figure 21: Spatial Analysis of Third-Class Health Centers

LINKS TO ATTACHED FILES OF GEOHEALTH

1. https://forms.gle/44rDwLGu4L5xuzwB8
2. https://drive.google.com/drive/folders/1iC17q_tbGlvKzKZ9nMVDeoO2RVSdGipu?usp=sharing
